# Supplementary material for: Seminoma and Embryonal Carcinoma Footprints Identified by Analysis of Integrated Genome-Wide Epigenetic and Expression Profiles of Germ Cell Cancer Cell Lines
Source: PLoS One. 2014 Jun 2;9(6):e98330. doi: 10.1371/journal.pone.0098330 (PMC4041891; doi:10.1371/journal.pone.0098330)
Supplement: File S1 — ZIP file containing DMRforPairs output for significant regions. Please start from the html files. (ZIP) [file pone.0098330.s008.zip › figures/21552.pdf]

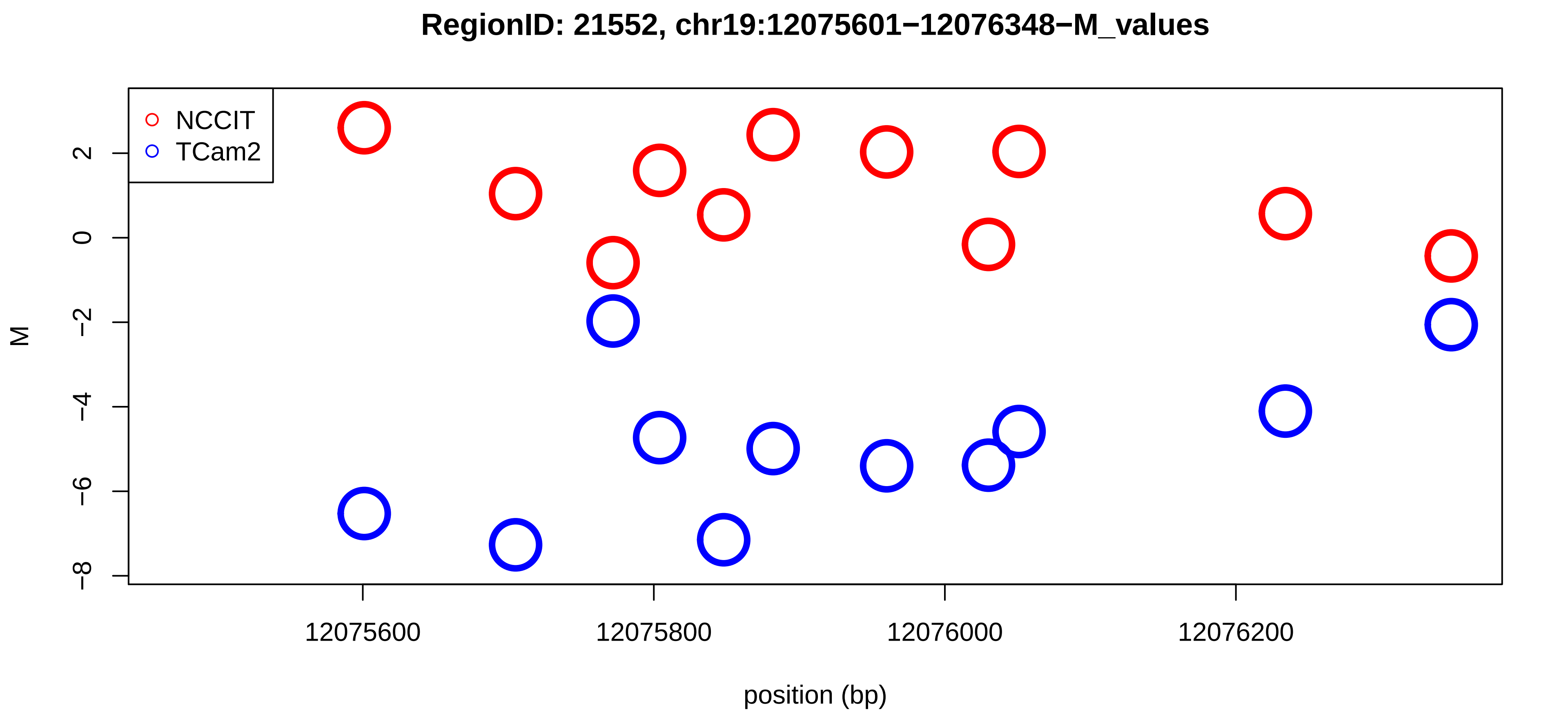
RegionID: 21552, chr19:12075601−12076348−M_values
NCCIT
TCam2
M
2
0
−2
−4
−6
−8
12075600
12075800
12076000
12076200
position (bp)

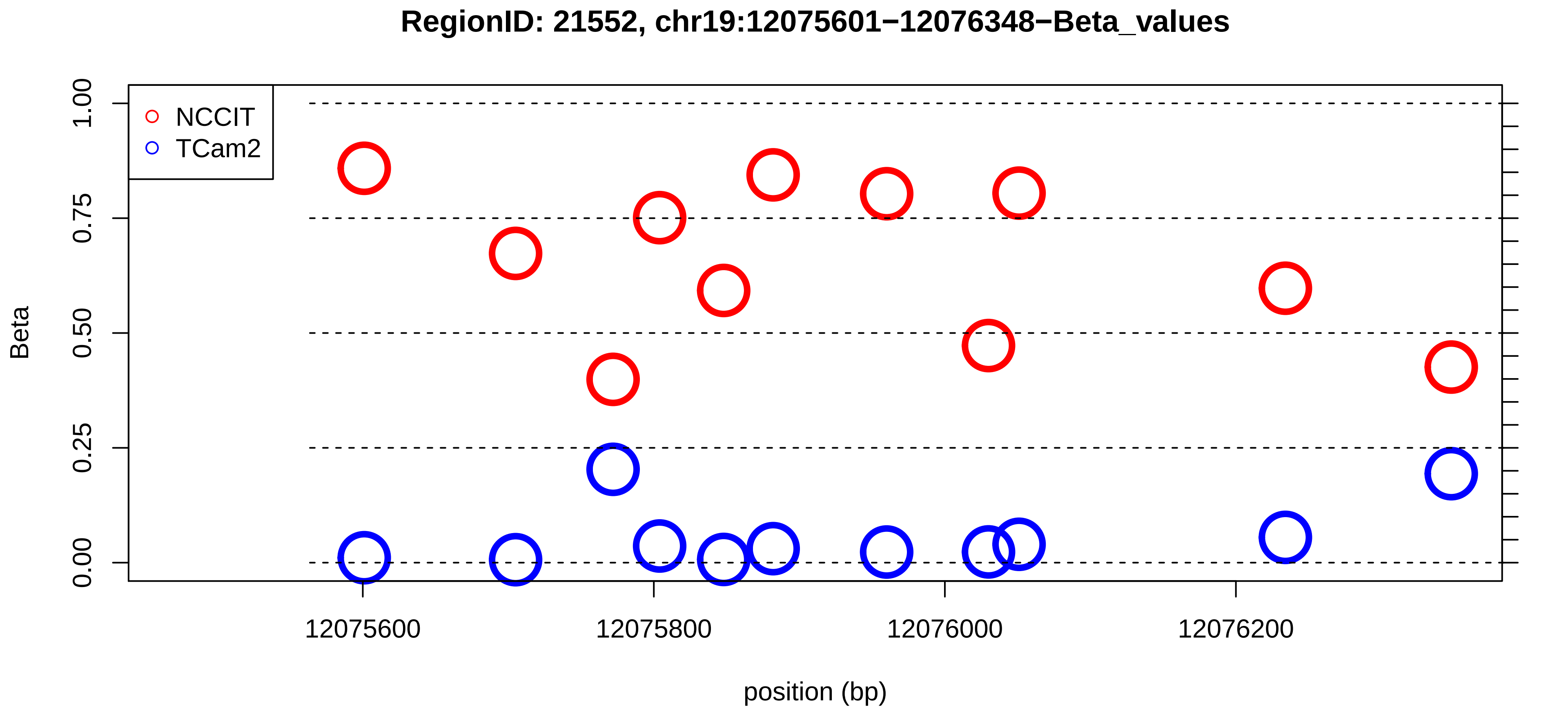
RegionID: 21552, chr19:12075601−12076348−Beta_values
NCCIT
TCam2
Beta
1.00
0.75
0.50
0.25
0.00
12075600
12075800
12076000
12076200
position (bp)

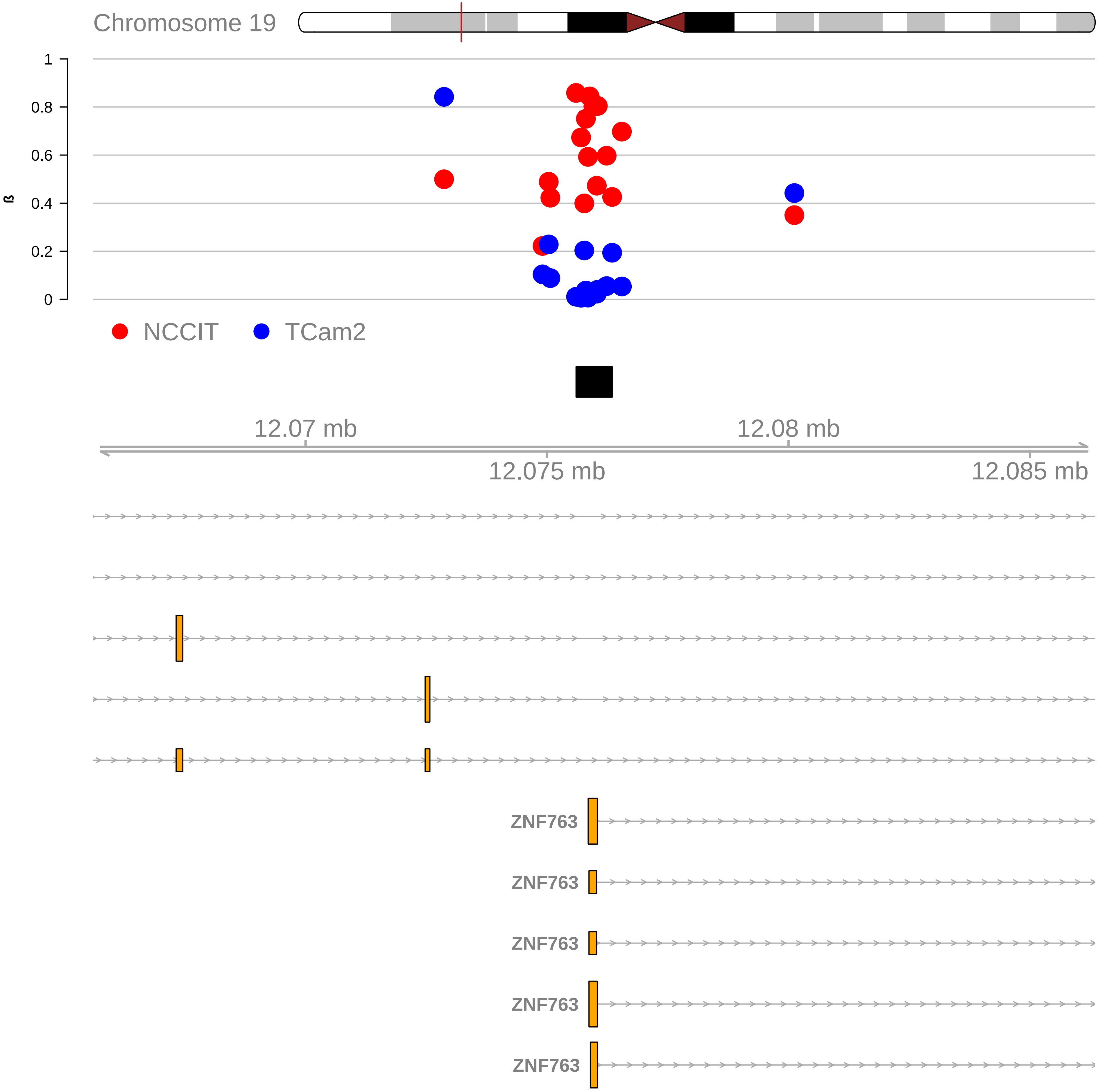

Chromosome 19
1
0.8
0.6
0.4
0.2
0
ß
NCCIT
TCam2
12.07 mb
12.08 mb
12.075 mb
12.085 mb
ZNF763
ZNF763
ZNF763
ZNF763
ZNF763
